# Deep learning for MRI lesion segmentation in rectal cancer

**DOI:** 10.3389/fmed.2024.1394262

**Published:** 2024-06-25

**Authors:** Mingwei Yang, Miyang Yang, Lanlan Yang, Zhaochu Wang, Peiyun Ye, Chujie Chen, Liyuan Fu, Shangwen Xu

**Affiliations:** ^1^Department of General Surgery, Nanfang Hospital Zengcheng Campus, Guangzhou, Guangdong, China; ^2^Department of Radiology, Fuzong Teaching Hospital, Fujian University of Traditional Chinese Medicine, Fuzhou, Fujian, China; ^3^Department of Radiology, 900th Hospital of Joint Logistics Support Force, Fuzhou, Fujian, China

**Keywords:** rectal cancer, magnetic resonance imaging, deep learning, lesion segmentation, review

## Abstract

Rectal cancer (RC) is a globally prevalent malignant tumor, presenting significant challenges in its management and treatment. Currently, magnetic resonance imaging (MRI) offers superior soft tissue contrast and radiation-free effects for RC patients, making it the most widely used and effective detection method. In early screening, radiologists rely on patients’ medical radiology characteristics and their extensive clinical experience for diagnosis. However, diagnostic accuracy may be hindered by factors such as limited expertise, visual fatigue, and image clarity issues, resulting in misdiagnosis or missed diagnosis. Moreover, the distribution of surrounding organs in RC is extensive with some organs having similar shapes to the tumor but unclear boundaries; these complexities greatly impede doctors’ ability to diagnose RC accurately. With recent advancements in artificial intelligence, machine learning techniques like deep learning (DL) have demonstrated immense potential and broad prospects in medical image analysis. The emergence of this approach has significantly enhanced research capabilities in medical image classification, detection, and segmentation fields with particular emphasis on medical image segmentation. This review aims to discuss the developmental process of DL segmentation algorithms along with their application progress in lesion segmentation from MRI images of RC to provide theoretical guidance and support for further advancements in this field.

## 1 Introduction

Colorectal cancer (CRC) is one of the most common malignant tumors in the digestive system worldwide. According to the Global Cancer Statistics 2018 released by the World Health Organization, an estimated 1.8 million new cases of CRC and 861,000 deaths were reported in 2018. Colorectal cancer ranked third in terms of incidence (constituting approximately 10.2% of all cancer cases) and second in terms of mortality (accounting for around 9.2% of all cancer-related deaths) (1). The incidence rate is higher in developed countries and regions. Among them, Rectal cancer (RC) is a prevalent malignancy worldwide, ranking second in incidence among all gastrointestinal tumors and representing the third leading cause of global cancer-related mortality (2). Accurate diagnosis and treatment of RC are pivotal in enhancing the long-term survival outcomes for patients (3). Currently, as a result of the widespread implementation of early detection methods for RC and continuous advancements in medical imaging technology, an increasing number of patients with RC can be identified at an early stage and receive optimal treatment (3, 4). As a pivotal imaging modality in the field of radiology, magnetic resonance imaging (MRI) proficiently delineates tumor morphology and precise localization, lymph node staging, extramural vascular invasion, as well as rectosigmoid mesentery fascia involvement (5). It has emerged as the foremost choice for diagnosing RC (6–8). However, the conventional radiology diagnosis of RC often necessitates doctors with extensive diagnostic expertise. Typically, radiologists are required to meticulously examine MRI images frame by frame, and accurately annotating the lesion area at a pixel level poses a significant challenge for physicians when determining the target region for radiation therapy in RC patients (9). Simultaneously, the substantial patient volume encountered in clinical practice significantly exacerbates their workload. Prolonged and repeated repetitive image analysis can potentially lead to misdiagnosis and failure to detect certain conditions, thereby impeding timely treatment initiation for these patients (10). The MRI images of RC often pose the following diagnostic challenges: ① There is considerable interindividual variability in the size and shape of RC, while the pelvic region exhibits complex anatomical structures. ② The region of interest (ROI) occupies a relatively small proportion within the image, certain organs exhibit analogous morphologies to the RC and are situated in close proximity, resulting in indistinct boundaries of RC and rendering diagnosis and differentiation challenging. Therefore, the development of a precise segmentation algorithm for the MRI images of RC is imperative to alleviate the burden on healthcare professionals and enhance the accuracy of diagnosis as well as efficiency in radiotherapy planning through computer algorithm-driven automatic identification of lesions associated with RC in MRI images.

Deep learning (DL), as a fundamental technology in the new era of artificial intelligence, enables the construction of highly effective machine learning algorithms based on extracted features. Integration of this algorithm with computer-aided diagnosis (CAD) technology not only eliminates subjective human factors but also facilitates accurate and efficient processing of massive medical data by clinical practitioners. Currently, DL-based CAD systems have been extensively employed in diverse medical image processing applications and have exhibited remarkable efficacy (11–13). Furthermore, the storage format of medical images adheres to the globally recognized DICOM (Digital Imaging and Communications in Medicine) standard, which serves as a robust foundation for the advancement of DL due to its inherent advantages such as universality, standardization, and exceptional quality. Currently, this technology has gained widespread application in the preoperative TNM staging of RC, assessment of neoadjuvant therapy efficacy, lesion segmentation, and non-invasive preoperative prediction combined with genetic typing (14–17). DL-based segmentation algorithms are end-to-end structures, where after the model architecture is completed, radiologists only need to focus on the input and output ends of the model during training and application. This eliminates the need for adjusting algorithmic encoding rules and optimizations based on intermediate results as required by traditional segmentation algorithms, thereby significantly enhancing work efficiency and facilitating practical clinical implementation. The DL-based segmentation algorithms currently achieve outstanding performance, surpassing various publicly available computer vision benchmark datasets and being widely applied in medical image processing (18, 19). Although there have been many studies on RC segmentation algorithms based on DL, there has not been a comprehensive review summarizing previous literature. The objective of this review is to present a comprehensive overview of the developmental process related to MRI-based DL segmentation algorithms, as well as the current research status in RC for image lesion segmentation. The ultimate aim is to provide more systematic guidance for advancements in this field.

## 2 Commonly DL-based algorithm for image semantic segmentation

**Figure 1:**
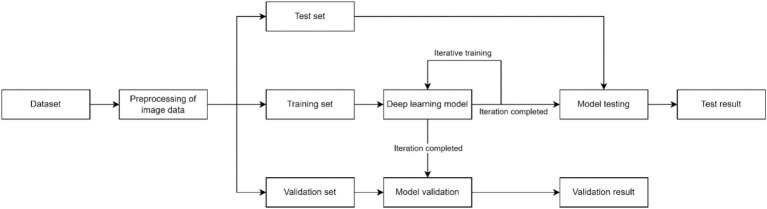
Flowchart of DL.

The concept of DL, initially introduced by the esteemed machine learning expert Hinton in 2006, represents a prominent form of machine learning (20). The core of DL lies in constructing machine learning architectures with multiple hidden layers, training them on large-scale datasets, and extracting a substantial amount of representative feature information to achieve accurate sample classification and prediction (21). The workflow typically encompasses three stages: ① preprocessing of image data; ② training, validation, and testing of the model; and ③ evaluation of the model (22). The preprocessing of image data is a fundamental task in DL, encompassing noise reduction, data normalization, feature selection, and extraction (23). To enhance model training and optimize accuracy, we typically partition them into three distinct subsets: the training set, validation set, and test set. The training set facilitates data parameter learning for classifier fitting, while the validation set serves as a safeguard against overfitting. Subsequently, the test set is employed to assess model performance. Ultimately, model evaluation is conducted to ascertain whether the research objectives are effectively achieved. Figure 1 presents a comprehensive flowchart illustrating the principles of DL.

Currently, computer vision encompasses various subtasks, including image classification, image segmentation, object detection, image annotation, and image generation. Among these tasks, image segmentation plays a pivotal role in medical image processing by facilitating the extraction of annotated ROI from 2D or 3D images. This technique generates a mask image with identical dimensions to the original image, where pixels representing ROI are assigned specific values (e.g., 0 for background region and 1 for ROI), thereby indicating the results of segmentation (24). The conventional image segmentation algorithms can be broadly categorized into several groups, encompassing threshold-based segmentation algorithms, edge-based segmentation algorithms, region-based segmentation algorithms, and clustering-based segmentation algorithms (25). However, these algorithms are relatively simplistic, primarily relying on elementary features such as texture and shape of the image, while disregarding the distinctions between diverse objects. The DL-based algorithms for image semantic segmentation leverage the exceptional feature learning capabilities of neural network models, enabling them to effectively capture and model the intricate semantic information as well as the interdependencies between various regions within images. This remarkable advancement has surpassed traditional image segmentation approaches, thereby showcasing its immense potential for further advancements in this field.

### 2.1 Convolutional neural networks

The Convolutional Neural Networks (CNN) serve as the predominant algorithmic models in DL applications, being fundamentally embraced as the foundational network for contemporary medical image segmentation algorithms. Although the concept of CNN was initially proposed by Fukushima et al. in the 1980s, and recognition based on receptive fields was invented to simulate the human visual system, research related to CNN faced significant limitations due to scarce computer hardware resources and insufficient training data at that time (26). Krizhevsky et al. developed AlexNet for the ImageNet Large Scale Visual Recognition Challenge (ILSVRC) until 2012, resulting in a substantial enhancement of image classification accuracy from 70 to 80% compared to conventional algorithms. This breakthrough prompted a resurgence of interest among researchers in the field of CNN (27). Subsequently, a plethora of seminal CNN models such as VGGNet, ResNet, GoogleNet, and DenseNet emerged in rapid succession (28–31). These models have found extensive applications across diverse image-processing tasks and have even surpassed human cognitive capabilities in certain aspects.

**Figure 2:**
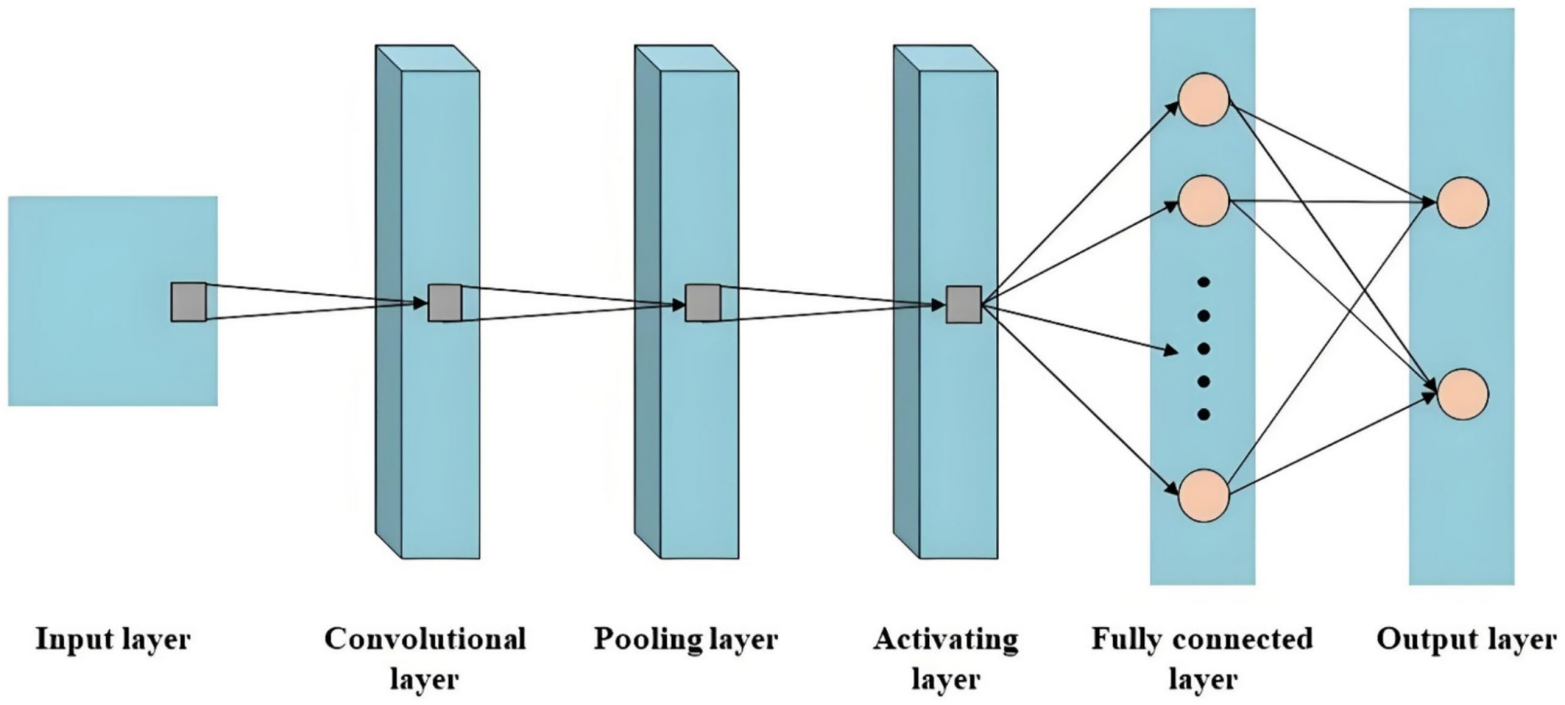
The schematic diagram of CNN.

CNN, developed based on traditional artificial neural networks, plays a pivotal role in the implementation of DL techniques for image recognition (32). The fundamental architecture is illustrated in Figure 2, comprising five distinct components: the input layer, convolutional layer, pooling layer, activating layer, fully connected layer, and output layer (33, 34). Firstly, the image is transmitted to the input layer in the form of a 3D pixel matrix, where the dimensions of the matrix represent the size of the image, and its depth represents the number of color channels. The convolutional layer automatically extracts high-level features that are relevant to accomplishing the given task. The pooling layer sparsely processes input feature maps to effectively reduce computational load. Subsequently, through an alternating stacking of convolutional and pooling layers, features are extracted and analyzed by the fully connected layer acting as a classifier for specific task classification. Finally, probabilistic scores for corresponding categories are provided by the output layer.

**Figure 3:**
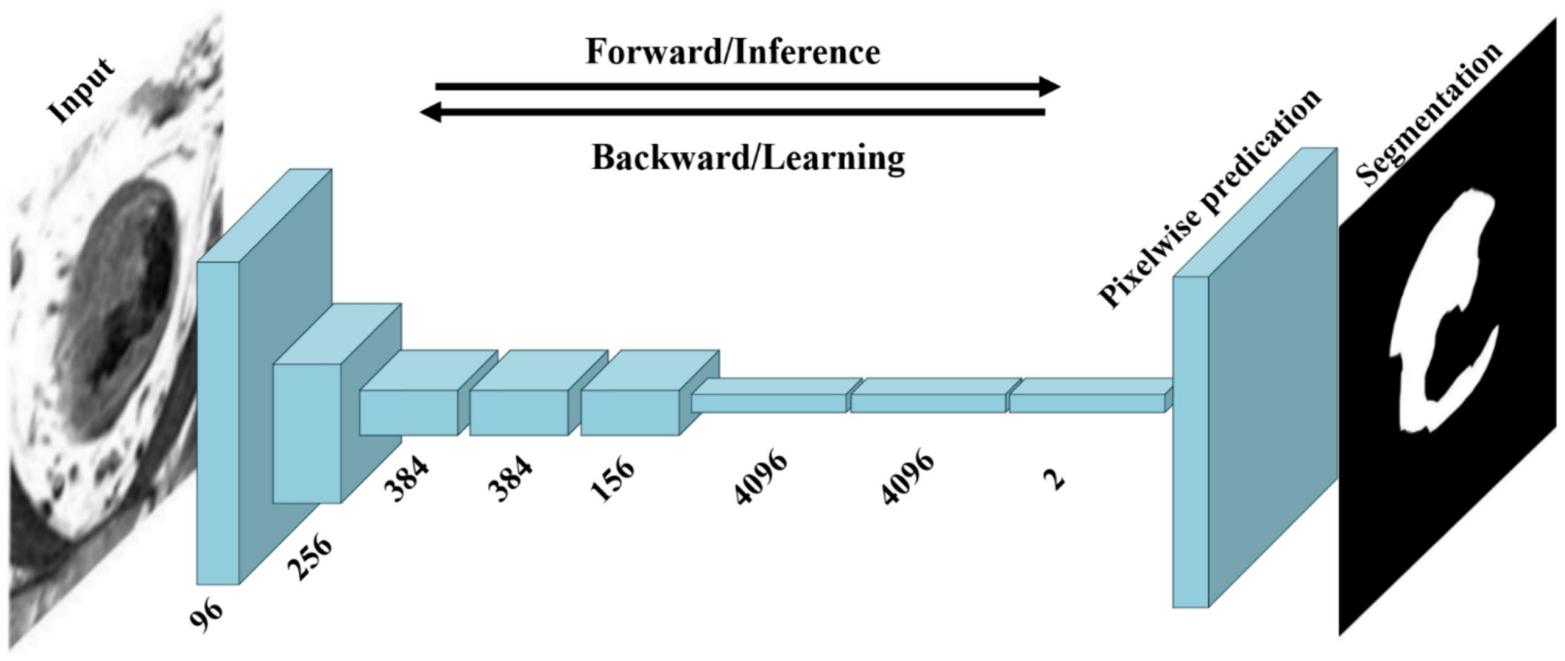
The schematic diagram of FCN.

Early CNN models employed fully connected layers at the final stage, leading to the loss of spatial information inherent in the input image. Consequently, these models encountered challenges in accurately determining the affiliation category for each pixel within the input image. In order to tackle this challenge, Long et al. introduced the Fully Convolutional Network (FCN) in 2015 and pioneered the application of CNN in the domain of image segmentation (35). The VGG Net and Inception Net models were employed as underlying structures for overlaying and conducting deconvolution operations on feature maps generated by various convolutional modules, resulting in segmentation outcomes that maintain consistency with the original image dimensions. As depicted in Figure 3, in contrast to conventional CNN, FCN exclusively comprises convolutional layer. In comparison with the input, convolution, pooling, fully connected and output processes of CNNs, the FCN procedure can be simplified into three steps: stacking alternating convolution and pooling layers, merging diverse layers, and performing up-sampling operations (35, 36). The advantages of FCN are as follows (37–39): ① The model eliminates a fully connected layer, effectively reducing the model’s complexity. ② By incorporating up-sampling operations that restore input feature maps’ resolution while preserving their original spatial information, FCN enables the use of images of any size as input, facilitating end-to-end pixel-level prediction. ③ FCN integrates skip connections to fuse feature maps from different levels, ensuring robustness and accuracy in predictions.

**Figure 4:**
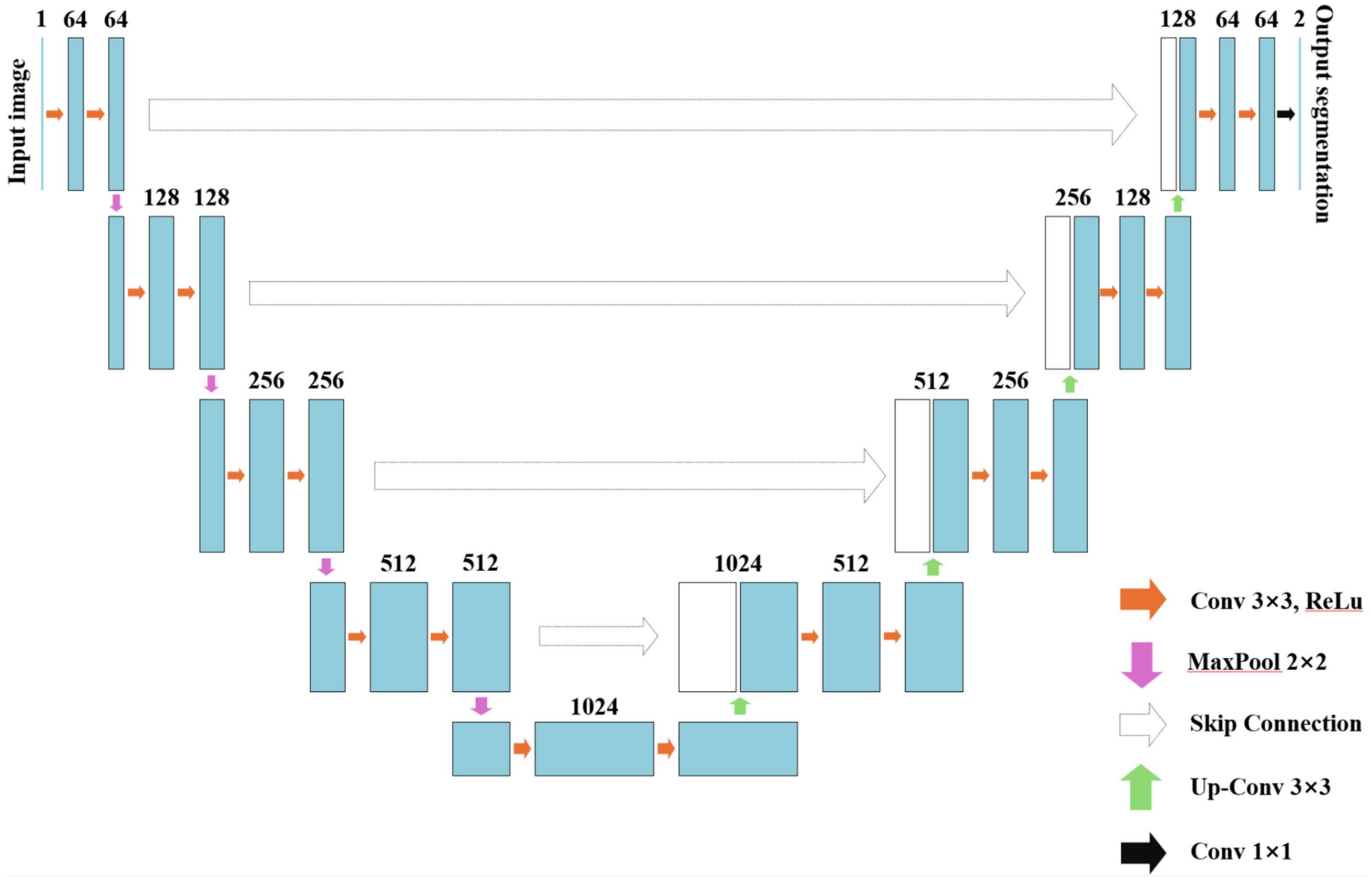
The schematic diagram of U-Net.

In the same year, Ronneberger et al. proposed U-Net, an FCN-based architecture designed for medical image datasets with limited samples (40). The major highlight of this model lies in its utilization of lateral skip connections within a symmetrical encoder-decoder architecture, facilitating the transfer of feature maps from the encoding process to the decoding process. This mechanism enables the fusion and complementation of low-level semantic information with high spatial resolution features, as well as high-level semantic information with lower spatial resolution features. By progressively enhancing the spatial resolution of encoder output features, it achieves seamless integration of high-level semantic information and high-resolution spatial details, thereby showcasing exceptional performance in medical image segmentation tasks (41, 42). The U-Net network is composed of two main components: the compression path and the expansion path. The compression path serves for feature extraction and aims to reduce the size of the feature maps. Each convolution block in the compression path consists of consecutive 3 × 3 convolutions, followed by a ReLU activation unit and a max pooling layer. This structure is iteratively applied multiple times. The distinctive characteristic of U-Net lies in its expansion path, where each stage employs a 2 × 2 deconvolution to upsample the feature maps. Subsequently, the upsampled feature maps are concatenated with their corresponding counterparts from the compression path through skip connections. Following this concatenation, two consecutive 3 × 3 convolutions and ReLU activation layers are employed. Finally, an additional 1 × 1 convolution is utilized to decrease the number of channels in order to generate segmented images as desired. By incorporating skip connections, U-Net effectively integrates low-level information with high-level features, enabling it to preserve more high-resolution details and further enhance accuracy in image segmentation. Consequently, this network has gained significant attention in the field of medical image segmentation and is widely employed as a primary model for various medical image segmentation tasks or as a benchmark model for evaluating model performance. The fundamental architecture of U-Net is illustrated in Figure 4.

Similar to the idea of skip connections in U-Net, SegNet was proposed by Badrinarayanan et al. in 2016 (43). The key contribution of the network architecture in this algorithm lies in its function as also an encoder-decoder, which stores index information during down-sampling pooling operations and utilizes these indices to recover corresponding information during up-sampling processes. In 2017, Zhao et al. introduced PSPNet, a novel approach that incorporates a pyramid pooling module to effectively integrate global contextual information with local semantic details, thereby augmenting the network’s capacity for scene understanding (44). The DeepLab network, proposed by Chen et al. in 2017, incorporated dilated convolution into the segmentation network to enhance the model’s receptive field. Additionally, fully connected conditional random fields were employed to refine the CNN-based segmentation results (45).

**Figure 5:**
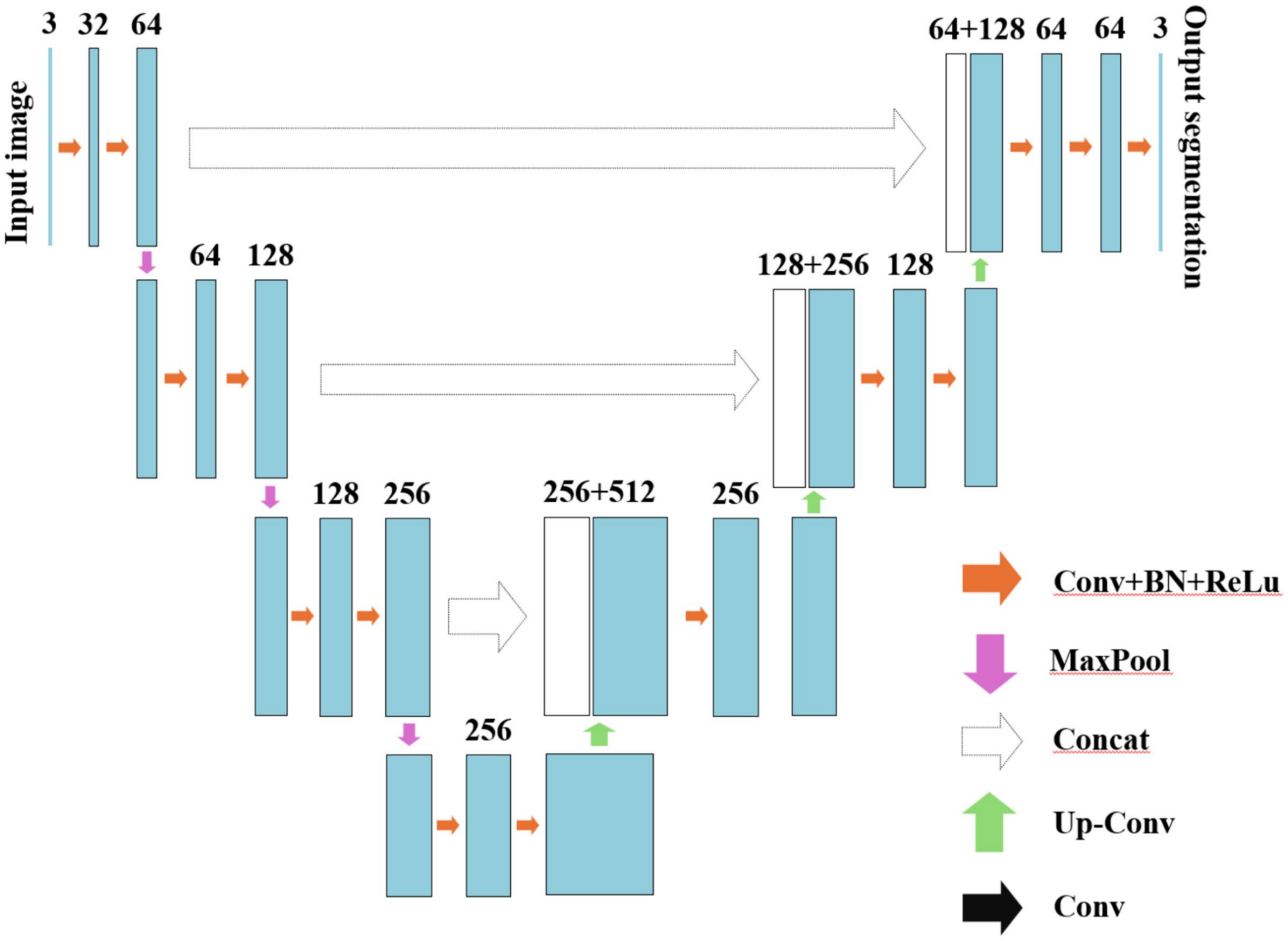
The schematic diagram of 3D U-Net.

Given the prevalence of 3D data in medical imaging, such as CT, MRI, PET, etc., there has been a proliferation of new 3D image segmentation algorithms within the realm of medical image segmentation, including notable examples like 3D U-Net (46). The 3D U-Net network model represents an enhanced iteration of the U-Net network, wherein all 2D operations have been substituted with their corresponding 3D counterparts, namely 3D convolution, 3D max pooling, and 3D deconvolution, resulting in 3D segmentation images (47). The fundamental architecture of 3 U-Net is illustrated in Figure 5, exhibits the capability to achieve image segmentation with minimal data owing to the abundance of repetitive structures and organizational information present in 3D images. Moreover, compared to its predecessors, this network demonstrates enhanced efficiency during the training process.

**Figure 6:**
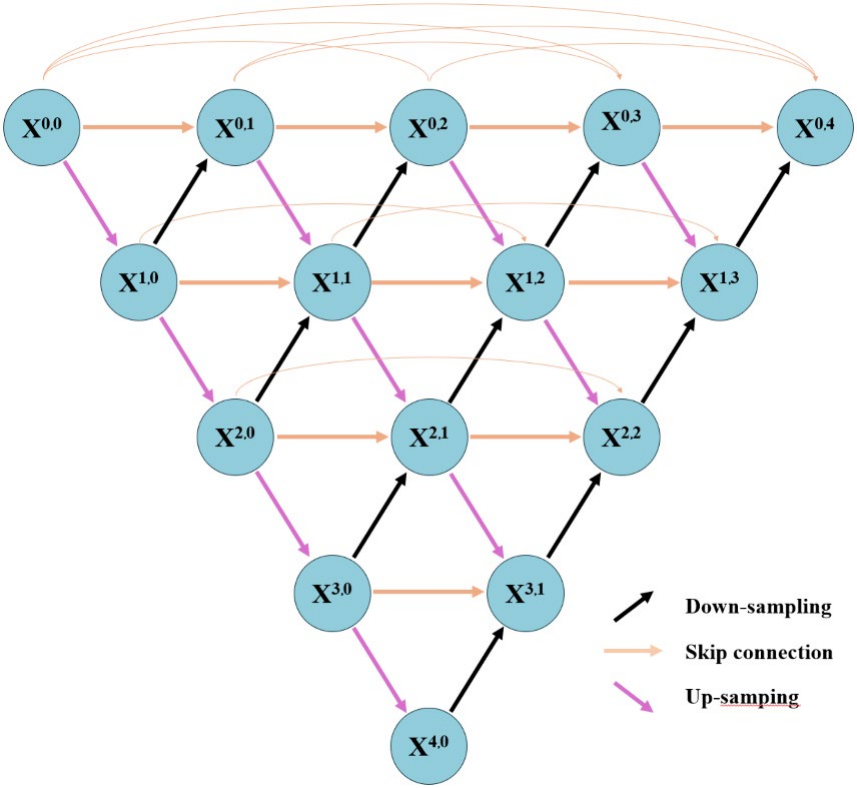
The schematic diagram of U-Net++.

Inspired by both DenseNet and U-Net, Zhou et al. proposed U-Net++, a potent variant based on the U-Net architecture (48). As depicted in Figure 6, U-Net++ employs dense skip connections to tightly link each convolutional block between the contracting and expanding paths, facilitating the preservation of more comprehensive semantic information throughout the network and enabling efficient image segmentation. In contrast to traditional U-Net where feature maps from the contracting path are directly connected to corresponding layers in the expanding path, U-Net++ introduces multiple skip connection nodes between each corresponding layer. Each skip connection receives feature maps from all nodes at the same level as well as directly upsampled feature maps from lower levels. This design the maximizes retention of semantic information between compression and expansion paths, resulting in enhanced segmentation performance.

The challenge in CNN-based semantic segmentation research lies in the loss of positional and detailed information during continuous pooling and extraction of high-level semantic features. This leads to incomplete restoration of such information during up-sampling, thereby impacting the accuracy of segmentation. The pooling function in SegNet (49), the skip connections between up-sampling and down-sampling in U-Net (41), 3D U-Net (46), and U-Net++ (48), and the fully connected conditional random field in DeepLab (45) are all designed to complement the detailed information during up-sampling operations. Moreover, integrating the semantic information extracted by CNN with local/global features is imperative for achieving accurate object segmentation across diverse scenes and varying sizes. The pyramid pooling module in PSPNet (50) and the dilated convolution in DeepLab (45) both synergistically fuse features from different spatial ranges to enhance segmentation efficacy. Currently, U-Net, 3D U-Net, and U-Net++ are widely recognized as classical neural network models in the field of medical image segmentation tasks.

### 2.2 Recurrent neural networks

The RNN is a pivotal component in the field of DL, extensively employed for processing time sequence data. The distinctive architecture characterized by self-connections within the hidden layers endows RNN with the ability to retain contextual information pertaining to temporal sequences. Owing to its unique internally recurrent structure, which sets it apart from other neural networks, RNN exhibits remarkable suitability for effectively handling sequential data (51, 52).

**Figure 7:**
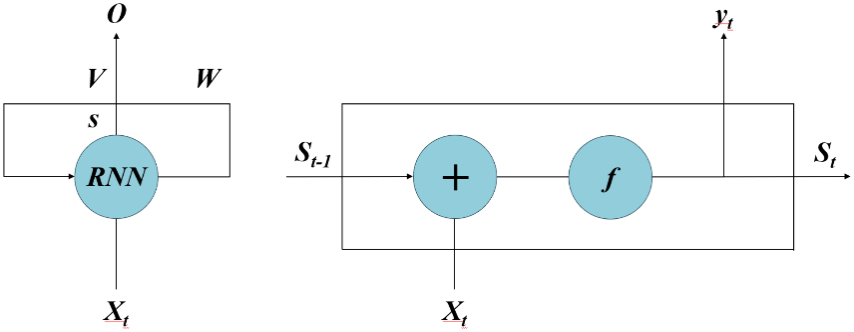
The schematic diagram of RNN.

The fundamental architecture of RNN is illustrated in Figure 7, which consists of an input layer, a hidden layer, and an output layer. This network exhibits a fully connected structure not only between layers but also within the hidden layer, enabling it to retain information from previous time steps and propagate it to subsequent ones. Consequently, the input of the hidden layer includes not only the input of the input layer but also the output of the previous time step’s hidden layer. The depth of an RNN manifests in two dimensions: vertical depth, allowing for multiple hidden layers to deepen network architecture; and horizontal depth, permitting multiple hidden layers in temporal dimension while retaining memory capabilities. As a result, RNN effectively handles sequential data features and achieves optimal predictive models (53, 54). *X_t_* represents the value of the input layer, *s* represents the value of the hidden layer, *W* represents the weight coefficient matrix when using previous hidden layer output as input for this time step, *o* represents the value of the output layer, *V* represents the weight coefficient matrix from the hidden layer to the output layer. For a given input *X* = (*X_1_*, *X_2_*,…, *X_n_*), by using formulas (1) and (2), we obtain a sequence of hidden layers *St* = (*S_1_*, *S_2_*,…, *S_n_*) and an output sequence *y_t_* = (*y_1_*, *y_2_*,…, *y_n_*) after passing through RNN.


(1) $$S_{t}=f\left(W_{ss}S_{t-1}+W_{xs}X_{t}+b_{s}\right)$$

(2) $$y_{t}=W_{sy}S_{t}+b_{y}$$

In this context, *W_ss_* denotes the weight coefficient matrix of the hidden layer, *W_xs_* represents the weight coefficient matrix from the input layer to the hidden layer, and *W_xs_* signifies the weight coefficient matrix from the hidden layer to the output layer. *b_s_* and *b_y, respectively,_* denote bias vectors of the hidden layer and output layer. The function *f* (*·*) represents activation functions such as sigmoid or tanh. The interconnected neurons in RNN’s hidden layer facilitate data sharing among neuron nodes, enabling effective handling of time series data.

**Figure 8:**
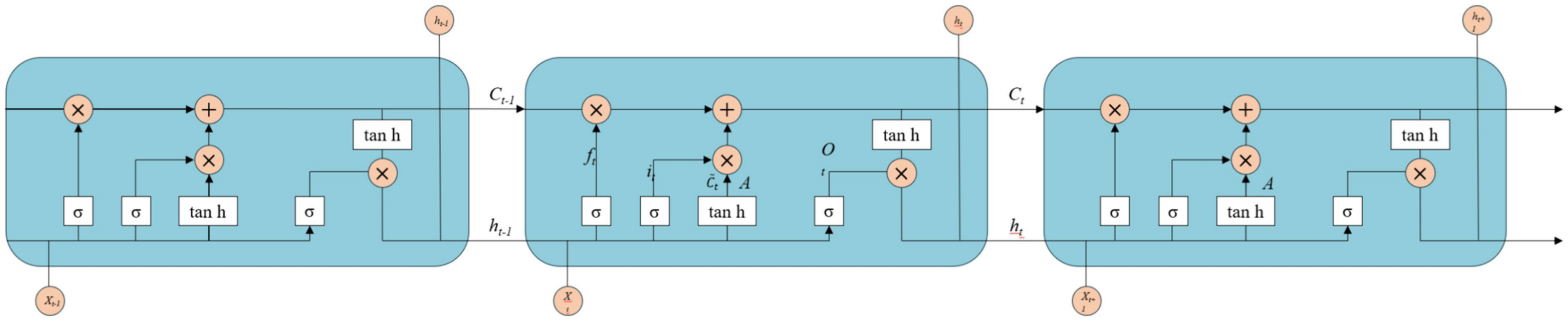
The schematic diagram of LSTM.

However, traditional RNNs face inherent challenges in addressing the issues of gradient vanishing and exploding during model training, which pose significant limitations on their application (55). To mitigate the problem of gradient vanishing in RNN, Hochreiter et al. proposed a novel Long Short-Term Memory (LSTM) neural network architecture (56). The LSTM incorporates three gates, namely the input gate, output gate, and forget gate, into the RNN. Upon information entry into the network, it undergoes evaluation based on predefined rules. Permissible information proceeds to subsequent steps while impermissible information is discarded via the forget gate. LSTM finds applications in diverse domains such as handwriting recognition, time series prediction, image analysis, and speech recognition. Currently, LSTM is extensively employed in the domains of handwriting recognition, time series prediction, as well as image and speech recognition (57). Gers et al. identified the limitations of the initial LSTM model and recognized the importance of periodically resetting the memory cell state and selectively forgetting irrelevant old information to accommodate new information storage during the process of information transmission (58). To address these issues, they introduced memory unit components on top of the original structure. The underlying design principle is that when stored content in the memory cell becomes irrelevant, it should be reset accordingly. This approach effectively mitigates both gradients vanishing and exploding problems while addressing long-term dependency concerns. Figure 8 presents a comprehensive flowchart illustrating the principles of LSTM.

In the LSTM architecture, the input information comprises of the current input state *X_t_* and the previous time step’s cell state *h_t − 1_*. The update mechanism involves filling or removing storage units within the internal structure. The gates in the LSTM design are constructed using a combination of sigmoid activation function and matrix dot product operations. The sigmoid activation function restricts its output values between 0 and 1, representing the extent to which information is allowed to propagate.

### 2.3 Graph neural networks

Although CNN and RNN have achieved decent results in some early diagnoses and image segmentation tasks, their limitation lies in the isolated extraction of individual imaging information, which hampers their ability to learn more effective models due to the inherent structure generated by predicting individual labels based on the interactions between partially labeled individuals and the entire population (59). The Graph Neural Network (GNN) framework has emerged in recent years as a powerful tool for directly learning from graph-structured data using DL techniques. Its exceptional performance has garnered significant attention and extensive exploration by researchers. By leveraging diverse types of information, including imaging and non-imaging data, GNN enhances the representation capability of individual subjects, enabling accurate prediction of individual labels based on interactions between partially labeled individuals and the entire population. Consequently, GNN finds wide application in fMRI disease diagnosis combined with population graph analysis (60). Bruna et al. were pioneers in the application of convolutional operations to GNN by leveraging a series of Laplacian operators, which enable a more direct representation of the convolutional properties in the Fourier domain of graph data (61). However, this approach is computationally intensive and overlooks local features. Defferrard et al. proposed ChebNet, a method that utilizes truncated Chebyshev polynomials to approximate spectral filters and avoid the need for computing Fourier bases (62). Kipf et al. introduced GGN with a local first-order approximation using spectral convolution (63). Currently, GGN employs a hierarchical propagation mechanism to encode node relationships from the graph structure as node features, thereby facilitating the generation of feature representations that encompass richer information. The GNN can be categorized into spectral-based approaches (62, 63) and spatial-based approaches (64, 65). Spectral-based GNN leverages the principles of spectral CNN, which are founded on graph Fourier transform and normalized Laplacian matrix. On the other hand, spatial-based GNN defines graph convolution operations based on the spatial relationships among graph nodes. However, as the number of graph convolution layers increases, there arises a phenomenon called ‘over-smoothing’ where high-level node representations tend to converge excessively. To address this issue and facilitate meaningful learning of high-level node representations, novel structures for GNN have also been proposed (66). The commonly used GNN structures are ChebNet (62), GCN (63), and JK-Net (66).

## 3 Application of DL based on MRI for lesion segmentation

Currently, the majority of research on RC tumor segmentation utilizing DL methods primarily focuses on imaging techniques such as T2WI, which enable the visualization of intricate anatomical structures. However, there is a paucity of studies investigating automatic segmentation algorithms for RC based on functional imaging modalities like DWI. Trebeschi et al. employed a DL model based on CNN to integrate T2WI + DWI (with B values of 1,000 and 0) images of RC patients, aligning the two image sets through deformable registration (67). However, suboptimal alignment between the two image sets may occur due to patient motion or involuntary bowel movement during scanning intervals. By exclusively utilizing DWI data for segmentation, potential errors in the registration process can be circumvented. Hence, it is imperative to investigate automatic segmentation of rectal tumors based on DWI. Irving et al. have developed an automated framework for tumor segmentation in RC patients using a superpixel approach and dynamic contrast-enhanced MRI (DCE-MRI) (68). This framework incorporates global anatomical morphological constraints to refine the boundaries of superpixels, resulting in excellent performance in DCE-MRI segmentation tasks. Moreover, this method can be extended to other DCE-MRI superpixel segmentation problems. Jian et al. utilized the complete rectal MRI image as input for the segmentation model and established five convolutional modules. Each module was capable of generating a corresponding predicted result map, which was subsequently fused to form the ultimate segmentation outcome of rectal tumors (69). Kim et al. employed a conventional U-Net architecture as the segmentation model, utilizing the entire rectal MRI image as input to automatically delineate both the rectum and tumor regions (70). Subsequently, they utilized the segmented output from this model as input for a classification network to determine the stage (T2 or T3) of the tumor in the rectal MRI image. Zhu et al. employed a fully supervised paradigm to train a 3D U-Net model on DWI images of 300 rectal cancer patients, resulting in a Dice coefficient segmentation score of 0.675 (71). These findings demonstrate the high accuracy and effectiveness of the DL model for tumor segmentation in DWI images of RC patients.

In rectal MRI images, the limited spatial coverage of rectal tumors poses a challenge for traditional CNNs to effectively capture both tumor-specific information and contextual details. Furthermore, the inclusion of hidden features surrounding the tumor is crucial for a comprehensive analysis of RC. To address this issue, some researchers have employed convolution kernels with varying sizes to extract features from the entire rectal MRI image, enabling simultaneous attention to subtle tumor characteristics and concealed features in its vicinity. The proposed multiscale convolutional architecture, as introduced by Men et al., employs VGG-16 as the underlying framework for accurate RC segmentation (72). By incorporating dilated convolutions at both the beginning and end of the main network, features at various scales in rectal images can be effectively extracted. Specifically, the initial dilated convolutions capture low-level contextual information while the subsequent ones capture high-level contextual information. Subsequently, Men et al. proposed a CAC-SPP model based on ResNet-101 for accurate segmentation of RC (73). This approach incorporates cascaded dilated convolutions and spatial pyramid pooling modules to effectively capture multi-scale features in rectal images, enabling the model to focus specifically on the contextual information surrounding rectal tumors.

In recent years, significant advancements have been made in the application of DL techniques for MRI image segmentation in RC. Presently, the primary focus within this field revolves around developing more efficient models utilizing innovative technologies. The utilization of U-Net architecture in DL has exhibited remarkable advancements in medical image segmentation tasks, positioning it as one of the prevailing focal points within this realm of scientific inquiry (16). The traditional U-Net network was enhanced by Li et al. through the introduction of a novel U-Net architecture (74). The proposed model introduces a novel approach by replacing the encoder with Squeeze-and-Excitation networks (SENet) and incorporating a global pooling layer after the last encoder. Additionally, spatial and channel compression is achieved through excitation attention mechanism modules in each decoder, followed by connecting the output results of each decoder. The research findings demonstrate that this model enables accurate and efficient RC segmentation as well as contour segmentation. DeSilvio et al. developed a U-Net model specifically designed for segmenting the rectal outer wall, lumen, and perirectal fat area in T2WI images after RC treatment (75). In a multi-institution evaluation, this region-specific U-Net achieved comparable performance to multiple radiologists in image segmentation tasks, with Dice coefficient indicators of 0.920 for bowel wall segmentation and 0.895 for bowel lumen segmentation (compared to radiologists’ scores of 0.946 and 0.873 respectively). Furthermore, this model exhibited a remarkable improvement of 20% over other types of U-Net models in terms of performance enhancement. The practical significance lies in its accurate assessment of tumor extent and precise delineation of rectal structures. Due to the limited ability of traditional U-Net networks to capture adequate contour information from extracted high-level features, a recent study by Dou et al. proposed an attention fusion U-Net model to enhance image segmentation accuracy (76). This model takes multi-parametric MRI images as input and effectively integrates their features through embedded attention fusion modules. Experimental results demonstrate that this approach achieves a Dice coefficient index of 0.821 ± 0.065 for segmentation, positioning it among the most advanced methods currently available for RC image segmentation.

## 4 Conclusion

DL based on MRI has demonstrated promising results in segmenting RC lesions and holds great potential for clinical applications. However, there is limited research specifically focused on MRI lesion segmentation for RC, with researchers primarily utilizing small-scale datasets that predominantly consist of T2WI MRI images. The investigation of lesion segmentation in other modalities of MRI, such as T1WI MRI images crucial for anatomical localization in clinical diagnosis, remains insufficient. Additionally, most existing modules in this field are designed for 2D image segmentation despite the fact that medical practice typically involves 3D MRI images. This approach may not accurately handle cases without tumor regions, leading to false segmentation issues. Furthermore, due to the relatively limited size of the test set used in this study, future research should encompass multicenter studies involving diverse medical centers and various types of MRI devices. Moreover, comprehensive exploration should be conducted on 3D convolutional segmentation models based on different modalities of MRI to provide robust technical support for precise localization of lesion positions during clinical diagnosis.

## Author contributions

MwY: Writing – original draft. MyY: Writing – original draft. LY: Writing – original draft. ZW: Writing – review & editing. PY: Writing – review & editing. CC: Writing – review & editing. LF: Writing – review & editing. SX: Writing – review & editing.
